# Accuracy of B-mode ultrasound and ARFI elastography in predicting malignancy of canine splenic lesions

**DOI:** 10.1038/s41598-022-08317-7

**Published:** 2022-03-11

**Authors:** Marjury Cristina Maronezi, Rafael Kretzer Carneiro, Igor Cezar Kniphoff da Cruz, Ana Paula Luiz de Oliveira, Andrigo Barboza De Nardi, Letícia Pavan, Priscila Del’Aguila-Silva, Ricardo Andrés Ramirez Uscategui, Marcus Antônio Rossi Feliciano

**Affiliations:** 1 Faculty of Agrarian and Veterinary Sciences, Paulista State University “Júlio de Mesquita Filho”, Jaboticabal, Brazil; 2 Universidad CES, Medellin, Colombia; 3 Federal University of Santa Maria, Av. Roraima nº 1000 Cidade Universitária Bairro - Camobi, Santa Maria, RS 97105-900 Brazil

**Keywords:** Diagnosis, Medical imaging, Prognosis

## Abstract

The objective was to evaluate the accuracy of B-mode ultrasonography and ARFI elastography in detecting malignancy in canine splenic lesions. Thirty-seven spleens with abnormalities (16 benign and 21 malignant) from dogs of different breeds and ages were evaluated. Echogenicity, echotexture, organ length and height were evaluated using B-mode. By ARFI elastography, tissue stiffness was evaluated qualitatively (elastogram) and quantitatively (measuring the shear wave velocity—SWV). Lesions were classified as diffuse, focal or multifocal (cranial, medial or caudal portion) and comparisons of the SWV between the injured and non-injured areas were performed. In the B-mode, no features were associated to malignancy (P > 0.05). In the elastogram, 35 spleens were non-deformable and 2 deformable, having no association with malignancy. The greater SWV was observed in malignant lesions (3.4 ± 0.6 m/s), followed by areas free from alterations (2.1 ± 0.3 m/s) and benign lesions (1.7 ± 0.5 m/s), with difference between groups (P < 0.0001). It was found that a SWV > 2.6 m/s indicates malignancy of canine splenic lesions (sensitivity of 95%, specificity of 100%, PPV of 100%, NPV of 94% and accuracy of 97%), concluding that ARFI elastography is a promising technique for differentiating malignancy in these lesions.

## Introduction

Splenic tumors have clinical relevance in the clinical routine of small animals, presenting a malignancy prevalence of 58% when patients have masses or nodules larger than 1 cm^1^. These animals may show nonspecific clinical signs such as weakness, anorexia, and lethargy^2^. Among the most diagnosed malignant and benign alterations in the spleen are hemangiosarcoma with an occurrence of 44.1% and nodular lymphoid hyperplasia affecting 20.1% of the animals^3^. The prognosis varies from favorable to reserved depending on the diagnosis of the splenic lesion^4^, which can be performed using ultrasound-guided fine-needle aspirate cytology or histopathological analysis of material collected by biopsies or splenectomy^5–7^.

In medicine, accurate and rapid diagnosis is essential for patient prognosis and therapy^8^. Ultrasonography is a sensitive technique to detect subtle alterations or abnormalities that affect the splenic parenchyma in small animals^7^, as it allows evaluating the organ in terms of size, contours, echogenicity, echotexture and identifying focal or diffuse lesions^9^. It is difficult to differentiate between benign and malignant processes using B-mode, as malignant and benign splenic tumors may show similar patterns of echogenicity and echotexture while tumors with the same histopathological result may show different echo patterns^10^.

Other ultrasound techniques such as Doppler and contrast-enhanced ultrasound (CEUS) have been studied as adjuvants in the differentiation of splenic lesions. CEUS showed no significant difference between malignant and benign parenchymal lesions in dogs^11^ as well as there was similarity in color Doppler and power Doppler between these lesions^12^. Thus, both methodologies, so far, cannot be used as predictors of malignancy in dog spleens.

Elastography provides complementary information to conventional ultrasound examinations, adding stiffness assessment as another measurable property. Among its modalities, the Acoustic Radiation Force Impulse (ARFI) is able to provide quantitative and qualitative information by generating shear waves^13^. ARFI has gained notoriety in veterinary medicine and medicine for demonstrating applicability in differentiating malignancy in thyroid^14^, cervical lymph nodes^15^, pancreas^16^ and mammary tumors^17^.

The quantitative evaluation of ARFI elastography is performed by measuring the shear wave velocity (SWV) and, through this technique, it has already been possible to differentiate benign from malignant lesions in different types of canine neoplasms (mammary, cutaneous and subcutaneous) and obtain cut-off values for this differentiation^17,18^. Also, in patients with breast cancer, it was possible to verify an increase in SWV in metastatic lymph nodes, differentiating from normal or inflamed tissues.

Believing that there are ultrasound and stiffness differences between malignant and benign splenic lesions in dogs, the present study aimed to evaluate the accuracy of B-mode ultrasound and ARFI elastography in detecting malignancy in splenic lesions in dogs. Our first hypothesis is that malignant tumors are more rigid than benign lesions, corroborating the results already found in other tissues. The second hypothesis is that benign tumors have greater rigidity when compared to normal splenic tissue.

## Results

In the present study, 37 spleens were evaluated, of which 10 (27%) had diffuse lesions, 6 (16%) lesions in the cranial region, 9 (24%) in the medial region and 12 (33%) in the caudal region. Of these lesions, 16 (43%) were classified as benign and 21 (57%) as malignant. The histopathological diagnosis of the lesions is shown in Table 1.

**Table 1:** Histopathological diagnosis and classification of splenic lesions in dogs previously submitted to the ARFI elastography evaluation.

Histopathological diagnosis	Classification	n	%
Splenic hemangiosarcoma	Malignant	10	27.0
Splenic hematoma	Benign	8	21.7
Complex lymphoid follicular hyperplasia	Benign	4	10.8
Lymphocytic lymphoma	Malignant	4	10.8
follicular lymphoma	Malignant	3	8.1
Epithelioid hemangiosarcoma	Malignant	2	5.4
Severe atrophy of white and red pulp	Benign	1	2.7
Splenosis	Benign	1	2.7
Splenic fibrosarcoma	Malignant	1	2.7
Splenic hemangioma	Benign	1	2.7
Extramedullary hematopoiesis	Benign	1	2.7
Poorly differentiated splenic sarcoma	Malignant	1	2.7
Total	–	37	100

*n* number.

The size of the spleen was similar in patients with benign and malignant lesions, and the height and length of the organ were not indicative of malignancy. Findings of echogenicity and echotexture were also similar between malignant and benign splenic alterations (Table 2).

**Table 2:** Association between B-mode ultrasound characteristics (height, length, echogenicity and echotexture) and malignancy of splenic lesions in dogs.

Variable	Classification	Benign	Malignant	P-comparation	P-diagnosis
Width (cm)	NA	5.9 ± 3.1	7.4 ± 3.6	0.2430	0.2442
Length (cm)	NA	5.0 ± 2.1	5.8 ± 3.2	0.4211	0.5604
Echogenicity	Anechoic	1/16 (6%)	1/21 (4%)	0.8732	–
	Hypoechoic	9/16 (56%)	10/21 (48%)		
	Mixed	6/16 (38%)	10/21 (48%)		
Echotexture	Heterogeneous	14/16 (88%)	20/21 (95%)	0.3523	–
	Homogeneous	2/16 (12%)	1/21 (5%)		

*NA* not applicable.

In the qualitative elastography evaluation, the spleen was classified as non-deformable in 35 (95%) and as deformable in 2 (5%) of the 37 examinations performed, however this classification is not related to the malignancy of the lesions (P = 0.465). Furthermore, there was a homogeneous stiffness pattern in 14 (88%) and heterogeneous in 2 (12%) of the 16 organs with benign lesions; in the 21 malignant lesions, the pattern was homogeneous in 16 (76%) and heterogeneous in 5 (24%). As with deformability, tissue stiffness homogeneity pattern was also not associated with malignancy (P = 0.2596).

The quantitative ARFI assessment of the different regions of the spleen was similar (P = 0.8856) between the cranial, caudal and medial portions and, in this way, the averages of each of these regions were calculated and used for the subsequent analysis, which were considered similar to each other in this general analysis (cranial 2.6 ± 0.8 m/s; medial 2.6 ± 0.9 m/s; caudal 2.5 ± 0.8 m/s; P = 0.9705).

Comparing the SWV from the lesion-free regions (2.1 ± 0.3 m/s) and the regions that presented focal or multifocal benign lesions (1.7 ± 0.5 m/s), it was observed that the free regions have a higher SWV (P = 0.0036). Focal or multifocal malignant lesions, on the other hand, had a SWV (3.4 ± 0.6 m/s) significantly greater than benign lesions and lesion-free areas (P < 0.0001).

In the analysis of the ability to predict malignancy through elastography, the anatomical regions in the organs that presented diffuse lesions were compared, and the medial region had the largest area under the curve (AUC = 0.9077) when compared to the cranial region (AUC = 0.8899) and the caudal region (AUC = 0.8661). For this reason, the SWV value of the medial area was used as the region of interest for the diagnosis of diffuse lesions.

**Figure 1:**
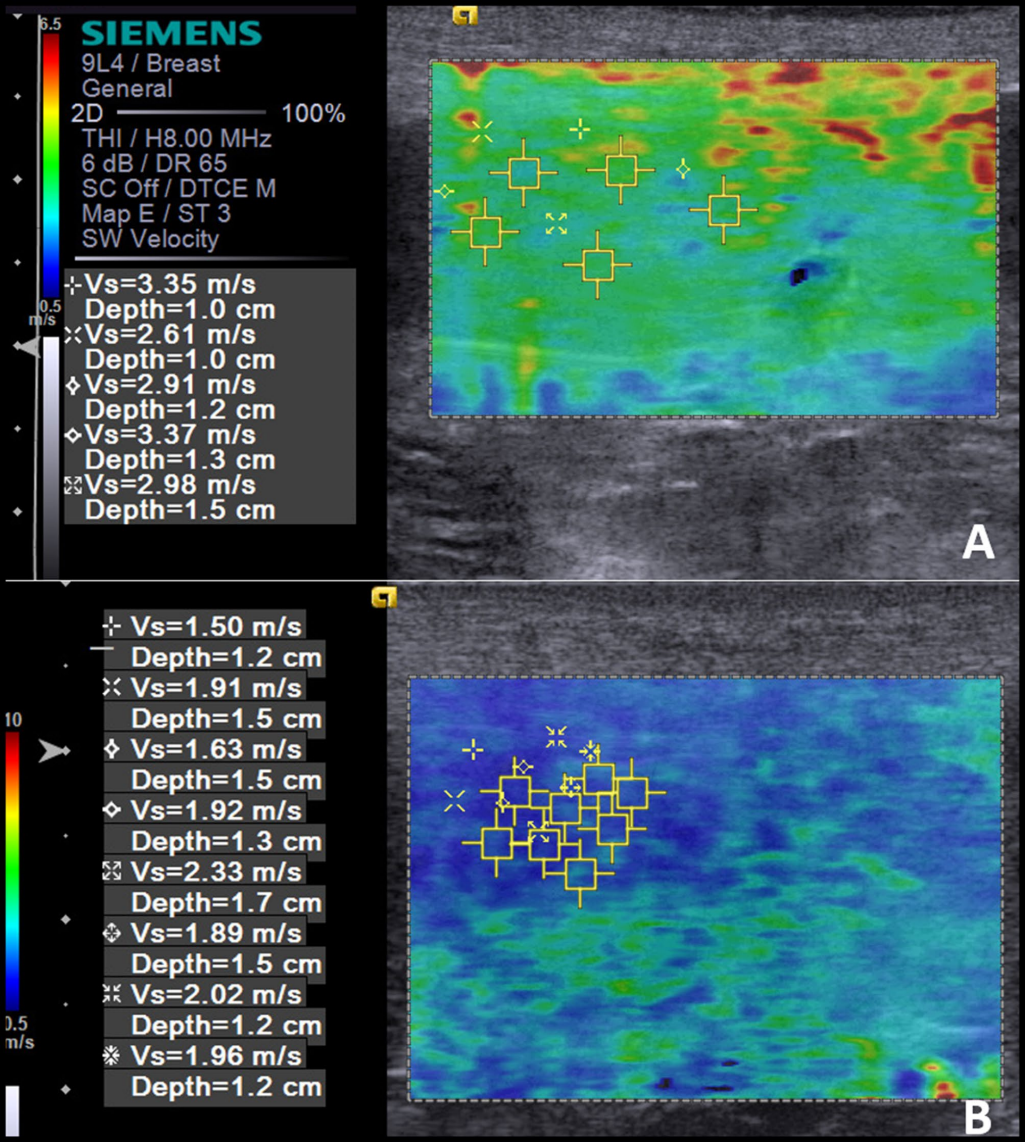
Ultrasonographic image of the spleen of two canine patients, using ARFI elastography (Virtual Touch Tissue Imaging and Quantification Elastography method). (**A**) Spleen with diffuse malignant alteration (multicentric lymphoma) presenting a mean shear velocity of 3.04 m/s; (**B**) Spleen with benign focal alteration (splenic hematoma) presenting an average shear velocity of 1.89 m/s.

Table 3 contains the mean values of the comparative descriptive statistical analyzes and the results of the analysis of the ROC curves to differentiate malignant lesions in the studied spleens. When comparing the SWV of the localized lesion regions and the medial region of diffuse lesions, it was possible to observe that in the benign lesions (1.7 ± 0.5 m/s) this variable is significantly lower (P < 0.0001) when compared to malignant lesions (3.4 ± 0.5 m/s), the discriminative power analysis indicated that a SWV > 2.6 m/s in focal lesions or in the medial region in diffuse lesions, indicates malignancy of the splenic lesion with a sensitivity of 95%, specificity of 100%, positive predictive value of 100%, negative predictive value of 94%, and an accuracy of 97%. The Fig. 1 illustrates the ARFI elastographic assessment in a benign (splenic hematoma) and a malignant (lymphoma) lesion. The standard deviation of these SWVs, as well as the depth of assessment, were similar in the different types of lesions and without any predictive power.

**Table 3:** Mean and standard deviation of shear wave velocity (SWV) for predicting malignancy in splenic lesions of dogs.

Variable	Benign	Malignant	P-Value	CP	Se%	Sp%	PPV%	NPV%	Acc%	AUC
SWV cranial	2.0 ± 0.5	3.0 ± 0.8	< 0.0001	> 2.6	71.4	93.8	93.8	71.4	81.1	0.8899
SWV Medial	1.9 ± 0.3	3.1 ± 0.8	< 0.0001	> 2.3	85.7	93.8	95.0	88.2	91.9	0.9077
SWV Caudal	2.0 ± 0.5	3.0 ± 0.7	< 0.0001	> 2.5	76.2	93.8	94.1	75.0	83.8	0.8661
SWV of the lesion and medial in diffuse	1.7 ± 0.5	3.4 ± 0.5	< 0.0001	> 2.6	95.2	100	100	94.1	97.3	0.9911
Standard deviation of SWV	0.3 ± 0.2	0.3 ± 0.2	0.4799	NA	NA	NA	NA	NA	NA	NA
Evaluation depth	2.1 ± 0.5	2.1 ± 0.6	0.8707	NA	NA	NA	NA	NA	NA	NA

*NA* not applicable, *CP* cutoff point, *Se* sensibility, *Sp* specificity, *PPV* positive predictive value, *NPV* negative predictive value.

## Discussion

The present study provided important information regarding the prediction of malignancy in splenic lesions of dogs using ARFI elastography. Malignant lesions presented greater tissue stiffness when compared to benign lesions, with a shear velocity (SWV) greater than 2.6 m/s, with a diagnostic accuracy of 97%. These results are important to determine a more adequate treatment and prognosis, as well as enabling a more agile therapeutic approach, in addition to these data being able to direct studies to other species.

The spleen is a reticuloendothelial organ^20^ and, due to its functional and anatomical characteristics, it is prone to develop neoplastic and non-neoplastic lesions^21^. Changes in the splenic parenchyma can be detected by conventional ultrasound^10^, however, this technique has low accuracy for differentiating malignancy from splenic changes^22^. In our study, there was no difference between the B-mode findings of malignant and benign lesions, which makes this method inconclusive, requiring more accurate techniques, such as cytology or histopathology or magnetic resonance^23^.

Ultrasound-guided aspiration puncture is considered invasive and complementary in splenic lesions, but reliable results for the diagnosis of abnormalities are often not obtained^7^. In contrast, histopathological examination is considered the gold standard for diagnosing these conditions as it is capable of providing accurate results^24^ and, therefore, it was the method of choice for diagnosing splenic lesions in this study. However, the collection of material for histopathology is more invasive and, in many cases, the delay in obtaining the diagnosis can retard the initiation of adequate therapy and, consequently, worsen the patient's prognosis^24^.

ARFI elastography proved to be a non-invasive technique capable of differentiating malignancy in different types of splenic lesions, with excellent predictive values and higher accuracy than other tests such as magnetic resonance which has 94% accuracy for differentiation between malignant and benign splenic lesions in dogs^23,25^.

Even if no association of qualitative characteristics (deformity and homogeneity) with malignancy of splenic lesions was observed, it is known that these assessments are evaluator-dependent^26^ and, consequently, are subject to different interpretations. However, in this study, it was possible to establish a significant difference in the shear velocity values of malignant and benign lesions, being an effective method to complement the study in B-mode, highly predictive of malignancy and that does not depend on the observer or the observations made and this so that it was possible to establish a cutoff value for the determination of malignancy (> 2.6 m/s).

The applicability of ARFI elastography has grown exponentially in recent years in medicine and veterinary medicine, so that this diagnostic modality can be used, in humans, as a predictor of malignancy in lymph node lesions^15,27^, in thyroid^28^ and splenomegaly^29,30^. Through this technique, it was already possible to verify that, in cases of breast cancer in bitches, metastatic lymph nodes had a higher shear wave velocity than healthy lymph nodes^31^, which is a promising technique in veterinary oncology.

In a previous study^29^, it was possible to verify differences in stiffness between spleens of healthy human patients, with hepatoportal, myeloproliferative and infectious diseases, with the control group having the lowest shear velocity, while the group with hepatoportal diseases had higher values for this measurement. This result was replicated^30^, demonstrating that the technique, in addition to being highly accurate for differentiating pathological processes, has good reproducibility of results.

Even though studies demonstrating the applicability of ARFI elastography in the study of splenic lesions in veterinary medicine have not been published so far, it has already been possible to differentiate malignancy between different mammary tumors of bitches, where malignant lesions were more rigid by ARFI elastography, with a sensitivity of 94.7% and a specificity of 97.2%^17^.

The increased stiffness observed in malignant splenic lesions in this study may be correlated with different pathological processes, such as type III collagen deposition, areas of fibrosis or microcalcifications^17^, while benign lesions, such as for example hematomas/clots, are basically formed by red blood cells and fibrin^32^, causing a decrease in this stiffness, making it even less rigid than in healthy tissue and free from injury.

The use of non-invasive tests without risk to patients and that allow for a differential diagnosis between malignant and benign lesions is of vital importance for animals and humans. Our results demonstrated that ARFI is useful in differentiating malignant and benign lesions in the spleen regardless of their location.

In conclusion, in this preliminary study the ARFI elastography made it possible to differentiate malignant and benign lesions in the spleen of dogs, and it was established that lesions with a shear wave velocity greater than 2.6 m/s are more likely to be malignant, with excellent predictive values. Thus, this imaging technique proved to be superior to conventional ultrasonography, which did not show satisfactory results for this differentiation.

## Methods

### Ethical aspects

All methods were performed in accordance with the relevant guidelines and regulations of the Brazilian National Council for the Control of Animal Experimentation (CONCEA) and were approved by the Ethics Committee in the Use of Animals of the São Paulo State University (Unesp), School of Agricultural and Veterinarian Sciences, Jaboticabal, São Paulo, Brazil (protocol number 014899/19) and follows the recommendations in the ARRIVE guidelines. Thirty-seven dogs of different breeds and ages were included, from the clinical routine of the Institution, during the years 2019 and 2020. For patient selection, the presence of splenic lesions identified prior to the B-mode exam was considered as an inclusion criterion.

### Ultrasound and elastographic evaluation

For the B-mode and elastographic examinations, a wide trichotomy of the abdominal region was performed and then the animals were positioned in dorsal and/or lateral decubitus. All exams were performed by the same experienced operator, without knowledge of the animal's clinical history and using an AcusonS2000® (Siemens®, Munich, Germany) ultrasonographic device with convex and linear multifrequency transducers (4.0–9.0 MHz).

In B-mode, the spleen was evaluated in transverse and longitudinal sections along its entire length, aiming to perform a complete tissue scan to identify the presence of lesions. Splenic alterations were classified as focal when there was a nodule in the splenic parenchyma, multifocal when there was more than one delimited area of lesion, or diffuse when there was change in the entire splenic parenchyma. Regarding ultrasonographic features, echogenicity (anechoic, hypoechoic, hyperechoic or mixed), echotexture (homogeneous or heterogeneous), contours/borders (regular or irregular) and organ size were evaluated, in addition to the size of focal lesions (length, width and length/width ratio). In diffuse lesions, the types (multiple circumscribed or disseminated alteration), quantity (when quantifiable) and size of the lesions (length, width and length/width ratio) were determined.

After the B-mode evaluation, ARFI elastography (qualitative and quantitative) was performed using the Virtual Touch Tissue Imaging and Quantification Elastography method, with the same ultrasound device. Qualitative ARFI provided color images (elastogram) that were evaluated through visual deformability (deformable or non-deformable), according to the shades of color observed, with bluish areas indicating soft or deformable tissues (elastic or not very rigid) and reddish shades indicating hard or non-deformable (rigid) tissues. The software itself features image quality control in which homogeneous greenish images indicated high quality of the technique and yellowish and heterogeneous images as low-quality technique. When inappropriate images were obtained, the exam was repeated.

For quantitative ARFI evaluation, tissue elasticity was automatically measured using the shear wave velocity (SWV) of the regions of interest (ROIs) defined by the operator by placing a 2.5 mm^2^ calipter on the elastogram images according to the following criteria: In spleens with one or more circumscribed lesions, at least three ROIs (those necessary to include the entire affected area) were selected from the abnormal area to obtain the mean SWV values, taking care to exclude vascular structures and cystic areas as described previously in evaluations of focal liver lesions in humans^32^. In addition, two ROIs were selected in each of the anatomical regions of the spleen (without apparent abnormalities): cranial extremity, body and caudal extremity to calculate the mean SWV of the apparently healthy tissue as already described in dogs^19^.

In spleens with diffuse lesions, a minimum of 12 ROIs were included, four in each of the previously defined areas of the splenic parenchyma (cranial, body and caudal), including the largest area of tissue and superficial, middle and deep subregions; for the calculation of the mean SWV of the spleen with diffuse lesion, taking care to exclude vascular structures, areas with a cystic, necrotic or calcified appearance as described by the consensus of the society of radiologists for the evaluation of diffuse liver lesions in humans^33^.

### Biopsy and splenic histopathological analysis

After ultrasound evaluation, tissue samples were collected, both from normal and altered areas, from all evaluated spleens. Ultrasound-guided incisional biopsy procedures with a trucut needle or surgical excisional biopsy under general anesthesia were performed, according to the protocol defined by the veterinarian responsible for the patient. The collected fragments were fixed in a 10% formalin solution, buffered with phosphate (pH 7.4) and processed until inclusion in paraffin and the prepared slides stained with hematoxylin/eosin. All samples were evaluated by the same experienced pathologist and were classified as malignant neoplastic lesion, benign neoplastic lesion or non-neoplastic lesion; focal or diffuse for each of the categories, totaling 6 types of classification.

## Data Availability

Statistical analysis was performed using the R software (R® Foundation for Statistical Computing, Vienna, Austria). Initially, the normal distribution (Shapiro–Wilk test) and the homoscedasticity of the variances (Barlett test) were tested. The real or transformed variables were then compared between the different ROIs by region, between regions, between free tissues, benign and malignant lesions, regions between diffuse lesions and medial region in diffuse lesions as well as affected regions between benign and malignant diagnosed by histopathological examination by Student's t-test. Subsequently, the parameters that showed significant differences (P ≤ 0.05) were submitted to discriminative power analysis (malignant lesions) through the Receiver Operating Characteristic Curves (ROC curve) and the cutoff point, sensitivity, specificity, positive predictive value, negative predictive value, accuracy and area under the curve (AUC) were calculated, using the logistic regression model.
